# Promoting Recruitment using Information Management Efficiently (PRIME): study protocol for a stepped-wedge cluster randomised controlled trial within the REstart or STop Antithrombotics Randomised Trial (RESTART)

**DOI:** 10.1186/s13063-016-1692-7

**Published:** 2017-03-01

**Authors:** Amy E. Maxwell, Martin Dennis, Anthony Rudd, Christopher J. Weir, Richard A. Parker, Rustam Al-Shahi Salman

**Affiliations:** 1 Centre for Clinical Brain Sciences, University of Edinburgh, Chancellor’s Building, 49 Little France Crescent, Edinburgh, EH16 4SB UK; 2 St Thomas’ Hospital, Westminster Bridge Road, London, UK; 3 Edinburgh Clinical Trials Unit and Centre for Population Health Sciences, Usher Institute of Population Health Sciences and Informatics, Medical School, University of Edinburgh, Teviot Place, Edinburgh, UK

**Keywords:** Methodology, Recruitment, Study within a trial, Stepped-wedge trial, Cluster randomised trial, Audit

## Abstract

**Background:**

Research into methods to boost recruitment has been identified as the highest priority for randomised controlled trial (RCT) methodological research in the United Kingdom. Slow recruitment delays the delivery of research and inflates costs. Using electronic patient records has been shown to boost recruitment to ongoing RCTs in primary care by identifying potentially eligible participants, but this approach remains relatively unexplored in secondary care, and for stroke in particular.

**Methods/design:**

The REstart or STop Antithrombotics Randomised Trial (RESTART; ISRCTN71907627) is an ongoing RCT of secondary prevention after stroke due to intracerebral haemorrhage. Promoting Recruitment using Information Management Efficiently (PRIME) is a stepped-wedge cluster randomised trial of a complex intervention to help RESTART sites increase their recruitment and attain their own target numbers of participants. Seventy-two hospital sites that were located in England, Wales or Scotland and were active in RESTART in June 2015 opted into PRIME. Sites were randomly allocated (using a computer-generated block randomisation algorithm, stratified by hospital location in Scotland vs. England/Wales) to one of 12 months in which the intervention would be delivered. All sites began in the control state. The intervention was delivered by a recruitment co-ordinator via a teleconference with each site. The intervention involved discussing recruitment strategies, providing software for each site to extract from their own stroke audit data lists of patients who were potentially eligible for RESTART, and a second teleconference to review progress 6 months later. The recruitment co-ordinator was blinded to the timing of the intervention until 2 months before it was due at a site. Staff at RESTART sites were blinded to the nature and timing of the intervention. The primary outcome is the total number of patients randomised into RESTART per month per site and will be analysed in a negative binomial generalised linear mixed model. PRIME began in September 2015. The last intervention was delivered in August 2016. Six-month follow-up will be complete in February 2017.

**Discussion:**

The final results of PRIME will be analysed and disseminated in 2017.

**Trial registration:**

The PRIME study was registered in the Northern Ireland Hub for Trials Methodology Research Studies Within a Trial (SWAT) repository (SWAT22) on 23 December 2015.

**Electronic supplementary material:**

The online version of this article (doi:10.1186/s13063-016-1692-7) contains supplementary material, which is available to authorized users.

## Background

Research into methods to boost recruitment into randomised controlled trials (RCTs) is the highest priority for trial methodological research. A Delphi survey of 48 UK Clinical Research Collaboration-registered clinical trials units achieved consensus, and it identified that the top three priorities for trial methodological research were ‘Research into methods to boost recruitment in trials’ (considered the highest priority), ‘Methods to minimise attrition’ and ‘Choosing appropriate outcomes to measure’ [1]. However, a systematic review concluded, ‘There is a clear knowledge gap with regard to effective strategies aimed at recruiters’ ([2], pg 1).

*The Lancet*’s series ‘Research: Increasing Value and Reducing Waste’ identified under-recruitment to RCTs as a major source of inefficiency in the conduct of applied clinical research [3]. Slow recruitment is particularly inefficient because it delays the delivery of research and inflates its costs by increasing the number of staff and sites or by extending the amount and duration of funding required. This problem has not been small; the authors of a review of 114 RCTs funded by the Medical Research Council (MRC) or the Health Technology Assessment programme in the United Kingdom for the period 1994–2002 found that less than one-third recruited their original target within the time originally specified, and around one-third were given extensions to achieve their target [4]. A marginal improvement was found when this review was repeated for the period 2002–2008; almost half of the RCTs did not recruit their originally specified target sample size, and nearly half of the RCTs received an extension of some kind [5]. Recruitment is jeopardised by many factors, including restrictive eligibility criteria and inefficient methods for approaching participants [6, 7].

The REstart or STop Antithrombotics Randomised Trial (RESTART [ISRCTN71907627]; http://www.restarttrial.org/) is an ongoing RCT comparing starting vs. avoiding anti-platelet drugs after stroke due to intracerebral haemorrhage (ICH) whose aim was to recruit 720 participants over 2 years, with recruitment rates estimated using epidemiological data [6, 8]. RESTART has implemented as many as possible of the strategies that have been shown to maximise recruitment [6, 9], and the chief investigator has used qualitative methods with principal investigators (PIs) at all active sites to identify and overcome barriers to clinician recruitment activity [10]. However, by the end of 2 years of recruitment in May 2015, 108 hospital sites were active in the United Kingdom and had recruited only 145 (20%) of the target number of participants in RESTART. Because there is no upper time limit on patient recruitment into RESTART after ICH onset, RESTART could boost recruitment by recruiting prevalent patients. Using electronic patient records has been shown to boost recruitment to ongoing RCTs in primary care by identifying potentially eligible participants [11, 12], but this approach remains relatively unexplored in secondary care, and for stroke in particular.

Therefore, we designed a stepped-wedge RCT embedded within RESTART to investigate whether recruitment into RESTART could be boosted by a complex intervention involving investigators in secondary care using electronic patient records held by national stroke audits. In this article, we report version 1.0 of the Promoting Recruitment using Information Management Efficiently (PRIME) work instruction to hospital sites, which constitutes the protocol for the trial.

## Methods/design

### Aims

The primary objective of the trial is to investigate if having a recruitment co-ordinator who conducts a recruitment review, provides access to bespoke stroke audit data exports, and conducts a follow-up review after six months improves the recruitment rate in RESTART at active hospital sites in the RESTART collaboration. The secondary objectives are to investigate the following questions:

- How many sites use routinely collected stroke audit data (or other data sources) to identify potentially eligible participants before the recruitment review?
- How many sites use the bespoke stroke audit data exports after the recruitment review, and how often?
- What are the barriers to recruitment in general?
- What are the barriers to using the bespoke stroke audit data exports?
- What are the disadvantages of using the bespoke stroke audit data exports?

### Design and setting

**Fig. 1:**
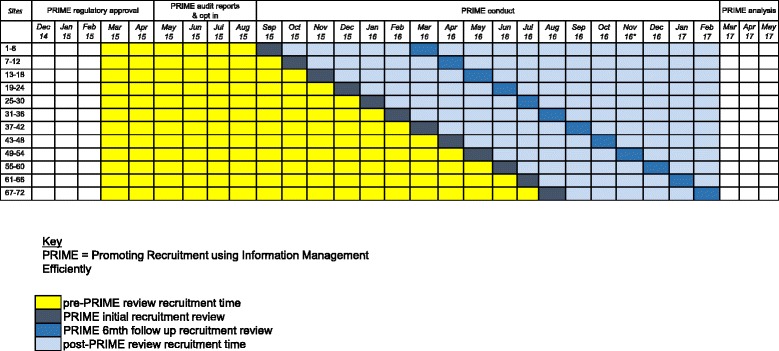
PRIME stepped-wedge randomised trial design. A schematic representation of the trial design

PRIME is a stepped-wedge cluster randomised trial (within the RESTART parallel-group RCT) investigating an intervention to boost recruitment in RESTART. At the time of inviting active hospital sites in RESTART to take part in PRIME in June 2015, there were 109 active hospital sites in the RESTART collaboration in the United Kingdom. We excluded 24 sites including two sites in Northern Ireland (where stroke audit data collection was not consistent), two sites in Scotland and two sites in England that piloted the PRIME intervention. We also excluded sites that had been activated to RESTART in 2015 to ensure that all sites taking part had a number of months of recruitment before receiving the intervention. The PRIME recruitment co-ordinator telephoned the RESTART co-ordinator at each of the remaining 85 sites in ascending order of site identification number to invite them to take part in a ‘recruitment review’, followed by an email if required, until 72 sites agreed. Sites were not informed about the exact content of the recruitment review or that its timing would be randomly allocated. The stepped-wedge design involves sequential roll-out of the intervention to clusters (active hospital sites in RESTART) over a number of time periods [13]. The month in which clusters start the intervention is randomly allocated so that groups of clusters begin the intervention sequentially at equally spaced time intervals (steps), and the outcome of interest is measured following each step until after all clusters have been allocated to the intervention (Fig. 1). A cluster randomised design was inevitable because the nature of the intervention meant that it could not be applied at the participant level. The stepped-wedge design is particularly relevant where it is predicted that the intervention will ultimately do more good than harm (making a parallel design, in which some clusters do not receive the intervention, potentially inefficient) and when the intervention cannot practically be delivered to all sites simultaneously. Because all clusters join the study at the start and are expected to remain until study end, the PRIME trial is an example of a closed cohort stepped-wedge design [14].

### Inclusion criteria

The inclusion criteria are as follows:

- Active RESTART site at the time of invitation for the intervention
- Site participates in data collection for the national stroke audits in Scotland or the rest of the United Kingdom
- Site is in Scotland, England or Wales

### Exclusion criteria

The exclusion criteria are as follows:

- Site participated in the PRIME pilot
- Site opted out when approached

### Randomisation

A senior programmer at the Edinburgh Clinical Trials Unit used a computer-generated block randomisation algorithm to randomly allocate the 72 sites into 12 strata of 6 sites each. Randomisation was stratified by hospital location (Scotland vs. England/Wales) to ensure that the proportions of sites with access to each national stroke audit data source were approximately consistent across the 12 groups. All sites began in the control state. The order in which the groups of sites step in to implement the intervention was determined by the randomisation algorithm. The randomisation list is held by the RESTART data manager.

### Blinding

The recruitment co-ordinator and staff at each participating site remained blinded to the timing of its randomly allocated ‘step’ until 2 months before the month allocated for each site’s PRIME intervention, when this had to be revealed in order to organise the recruitment review.

### The PRIME intervention

**Fig. 2:**
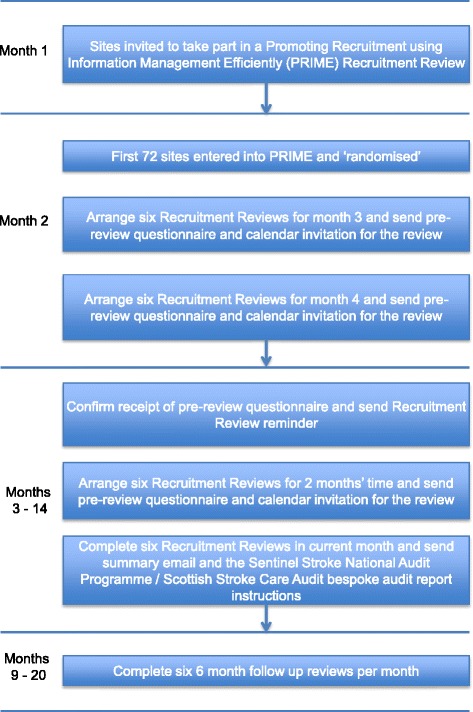
Trial flowchart. Standard Protocol Items: Recommendations for Interventional Trials (SPIRIT) figure displaying schedule of enrolment and interventions.

Two months before the allocated month of the recruitment review, the recruitment co-ordinator sent an email to RESTART collaborators at the site requesting that they provide suitable dates for their recruitment review teleconference, which would be held with the PI and the RESTART co-ordinator at the site (Fig. 2). Once a date is agreed upon, the RESTART site co-ordinator is sent a calendar invitation containing the dial-in details for the teleconference and a questionnaire to be completed before the review enquiring about (a) the main barriers preventing recruitment to RESTART, (b) what sources are used to identify potential participants and (c) what methods have been used to boost recruitment to RESTART (Additional file 1). One or two days before the recruitment review, a reminder of the dial-in details is sent, and if the pre-review questionnaire has not yet been completed and returned, it is re-sent with a further request to complete it before the recruitment review. Before the recruitment review, the recruitment co-ordinator records basic details about the site receiving the review, including (a) how long the site has been active; (b) how many patients have been recruited there; (c) whether they are part of the RESTART magnetic resonance imaging sub-study, and, if so, how many patients they have recruited into it; and (d) how many patients per year the site predicts they would recruit at their site initiation visit.

#### Recruitment review

The recruitment review consists of the following:

- Confirmation that all the relevant staff are present
- Review of RESTART delegation log to ensure it is up to date
- Discussion on how staff at the site have been finding RESTART
- Review of the recruitment commitment made at the site initiation visit and how many patients have been recruited
- Review of the data in the questionnaire about recruitment completed before the recruitment review
- Explanation of the availability of bespoke stroke audit data exports and examples provided of how the pilot sites effectively used them
- Review of the use of a template invitation letter for approaching prevalent patients and discussion about how they can be used effectively in conjunction with the bespoke stroke audit data exports
- Review of the opportunities to recruit inpatients and outpatients at the hospital site
- Sharing of what other methods top recruiting sites have been using to identify, consent and randomise patients
- Sharing any other relevant information about recruitment (e.g., presentations available on the RESTART website, collaborators meetings and poster presentations at conferences, telephone conferences with site staff)
- Confirmation that the site agrees to have a follow-up review in 6 months to review progress after the recruitment review

After the review, an email is sent to all the collaborators at the site, summarising what was discussed at the review, providing attendance certificates and giving instructions for running the relevant bespoke stroke audit data exports.

#### Bespoke stroke audit data exports

The data sources used in PRIME, alongside other local hospital databases, are the Scottish Stroke Care Audit (SSCA; http://www.strokeaudit.scot.nhs.uk/) and the Sentinel Stoke National Audit Programme (SSNAP; https://www.strokeaudit.org/Home.aspx). The SSCA was established in 2002 and includes all hospitals managing acute stroke in every Scottish NHS board, each of which uses the SSCA to evaluate its stroke care against national standards and to drive improvements; inpatient and outpatient data since January 2010 are available. The SSNAP has prospectively collected a minimum dataset for stroke patients in England and Wales since January 2013 to measure processes of acute care, rehabilitation, care in the community, and outcome measures at 6 months; inpatient data since January 2013 are available. Bespoke stroke audit data exports were created for PRIME in collaboration with the SSCA and SSNAP teams to reflect the RESTART eligibility criteria as best the routinely collected data can. The exports show hospital staff, who have access to stroke audit data, details about only their own hospital’s patients who might be eligible for RESTART. These data can be shared with local staff at each site on the RESTART delegation log who are authorised to also view information about patients within the clinical stroke service, with reference to local information governance procedures. Because the data will be exported locally, there will be no passage of identifiable, patient-level data to a third party such as the central RESTART trial co-ordinating team in Edinburgh. A set of instructions (Additional files 2 and 3) is provided to sites after their recruitment review, explaining how to produce the bespoke stroke audit data exports. The local research teams are responsible for validating the exported information, confirming any patient’s potential eligibility and contacting them to invite them to participate in RESTART using one of the template letters of invitation.

#### Six-month follow-up review

Two months before the 6-month follow-up review is due, an email is sent to determine availability. Once a date is agreed upon, the site is sent a questionnaire to be completed before the review, along with a calendar invitation containing the dial-in details for the teleconference. The questionnaire enquires about (a) whether they have run the bespoke stroke audit data exports since the recruitment review; (b) if so, how many of the patients identified were eligible; (c) how many eligible patients were recruited; and (d) whether the exports were useful (Additional files 4 and 5). One or two days before the follow-up review, a reminder of the dial-in details is sent, and if the questionnaire has not yet been completed and returned, it is re-sent with a further request to complete it before the review. The follow-up review consists of the following:

- A review of the use of the training given at the initial recruitment review
- A review of the yield of the bespoke stroke audit data exports
- Further recruitment tips not already provided at the initial recruitment review

### Comparator

All sites begin in a control state before receiving their recruitment review (Fig. 1). The control state period will vary from 6 to 17 months, depending on the month in which the site receives the recruitment review (i.e., the first component of the intervention). No recruitment review or any other intervention will be given to the sites during the control period. The control period will be used as a comparator for the analysis against the intervention period (which is 6–17 months after the site receives their initial recruitment review).

### Study outcomes

The primary outcome is the total number of patients randomised into RESTART per month per site. The RESTART trial database collects information about every randomisation in real time. The total number of people randomised per month will be quantified at each site for analysis. In PRIME, we will quantify the primary outcome at each site in the comparator period before the recruitment review occurs at that site, and we will measure the primary outcome in the intervention period after the recruitment review (Fig. 1). The time periods analysed are likely to be sufficient to allow enough time for the intervention to lead to recruitment of participants, and for us to be able to detect whether there is an initial surge in recruitment as a result of the recruitment review followed by a decay in the randomisation rate over time.

We will collect data on secondary outcomes from the information supplied in the questionnaires completed before, at and 6 months after the recruitment review to address the following subsidiary research questions:

- Number of sites in PRIME that routinely used stroke databases to identify potentially eligible patients before receiving the recruitment review
- Number of sites in PRIME that used the bespoke stroke audit data exports, and the frequency of their use in the 6 months after the recruitment review
- Barriers to recruitment in PRIME, identified by sites in the questionnaires completed before, at and 6 months after the recruitment review
- Barriers to using the bespoke stroke audit data exports identified by PRIME sites at the 6-month follow-up review
- Disadvantages of the bespoke stroke audit exports identified by PRIME sites at the 6-month follow-up review

### Statistical methods

We modelled our predicted recruitment to PRIME using live data from RESTART in March 2014 [15], when the rate of randomisation was 0.28 participants per site per month on average. RESTART recruitment data indicated that the standard deviation of the paired difference in rates within site before and after implementation of the intervention would be 0.75 (assuming the pessimistic scenario of independence between pre- and post-intervention rates within sites). Therefore, 72 sites would give 87% power to detect a change in rate of 0.28 participants per site per month (paired *t* test, two-sided 5% significance level). This would be consistent with the effect size that was seen in a previous before-and-after study in which researchers found that an electronic medical record-generated physician alert doubled the recruitment rate into an RCT [16].

This protocol and the statistical analysis plan will be submitted for publication before all sites receive the PRIME intervention and before outcome data are known. The statistical analysis plan will be published separately from this protocol. In brief, PRIME’s primary outcome of recruitment rate per site per month will be compared before and after the allocated month for the recruitment review using a negative binomial generalised linear mixed model, informed by a published method to model recruitment [17]. The primary analysis will follow an ‘as-randomised’ principle, which means that the data will be analysed according to the allocated timing of the recruitment review rather than the time that the recruitment review actually occurred. In addition, for the primary analysis, all sites will be included, regardless of any site withdrawals and/or compliance with the PRIME trial procedures. There will be no formal interim analyses or stopping rules for early termination, because PRIME is a trial of an intervention to manage the performance of sites to fulfil their recruitment targets and is not a trial on patients.

### Patient and public involvement

We have sought the views of the patient reference group for the Research to Understand Stroke due to Haemorrhage programme (www.RUSH.ed.ac.uk). No extra burden for patients was foreseen, given that the stroke audit data exports would be screened by each patient’s hospital team before each patient’s clinician invited them to participate. Attempting to recruit eligible, prevalent patients who had been discharged from hospital, but who might benefit from participation in RESTART, was seen to be a good thing. The patients reviewed and approved the wording of the letters that would be sent to invite prevalent patients to participate.

### Data protection

All PIs and study site staff involved with PRIME must comply with the requirements of the Data Protection Act 1998 with regard to the collection, storage, processing and disclosure of personal information, and all will uphold the act’s core principles and be appropriately trained in good clinical practice. Computers used to collate the data will have limited access measures via usernames and passwords.

### Roles and responsibilities

The day-to-day running of PRIME is conducted by the recruitment co-ordinator, supported and overseen by the RESTART trial manager and chief investigator. The progress of PRIME is overseen by the team that designed the trial (RASS, CJW, AR and MD) and is discussed at the main RESTART Trial Steering Committee meetings. There is no data monitoring committee, because there is no patient participation in this trial. RESTART is jointly sponsored by NHS Lothian and the University of Edinburgh (Academic and Clinical Central Office for Research and Development; http://www.accord.ed.ac.uk/). The sponsor and funder had no role in study design; collection, management, analysis and interpretation of data; the writing of this report; or the decision to submit the report for publication. The sponsor and funder will not have ultimate authority over any of these activities.

### Reporting

The trial will be reported in a manner consistent with the adaptation of the Consolidated Standards of Reporting Trials (CONSORT) reporting guidelines for cluster randomised trials [18], the guidelines for reporting embedded recruitment trials [19], and the recommendations for reporting stepped-wedge trials proposed by Hemming et al. [20] and Davey et al. [21]. The co-authors of the protocol will submit the final report for publication in a scientific journal and presentation at relevant conferences. Published results will not contain any personal data that could allow identification of individual participants.

## Discussion

PRIME is just one study within a trial (SWAT) amongst many others (http://www.methodologyhubs.mrc.ac.uk/resources/swat and http://www.qub.ac.uk/sites/TheNorthernIrelandNetworkforTrialsMethodologyResearch/SWATSWARInformation/) that are all attempting to improve the evidence base to optimise recruitment and retention in RCTs (e.g., http://www.trialforge.org/ and http://www.orrca.org.uk/). PRIME differs from many of these studies by using a cluster randomised design of an intervention at the site level rather than an intervention at the patient level, and using a stepped-wedge design to ensure that all sites receive the intervention in view of the investigators’ concern to ensure that all sites have the opportunity to boost their recruitment for the benefit of the parent RESTART trial.

PRIME encountered delays when it was reviewed by the study sponsor and the research ethics committee with oversight of the RESTART (parent) trial, despite the opinion that we obtained from the UK Health Research Authority. If the evidence base for the most efficient ways to conduct randomised trials is to be improved, regulatory agencies should consider creating policies and guidance for the review of methodological research embedded within randomised trials in order to standardise processes and facilitate low-risk methodological research. In the meantime, we recommend that future SWATs seek sponsor review and research ethics committee approval as early as possible.

The results of PRIME may be generalisable to future randomised trials of secondary prevention after stroke in the United Kingdom that could use electronic data held to identify potentially eligible participants. Results are expected to be analysed in 2017 and then published.

### Trial registration

The PRIME study is registered with the Northern Ireland Hub for Trials Methodology Research SWAT repository (SWAT22; http://bit.ly/2a4n7Yb) and was submitted to the Online Resource for Recruitment research in Clinical trials (ORRCA; http://www.orrca.org.uk/).

### Trial status

The first recruitment review took place in September 2015, and the last review in August 2016. The first 6-month follow-up took place in March 2016, with the last one in February 2017.

## Additional files

Additional file 1: Pre-recruitment review questionnaire. (PDF 409 kb)

Additional file 2: SSNAP RESTART reporting instructions. (PDF 609 kb)

Additional file 3: SSCA RESTART reporting instructions. (PDF 788 kb)

Additional file 4: Six-month follow-up review questionnaire (for PRIME sites using SSNAP). (PDF 396 kb)

Additional file 5: Six-month follow-up review questionnaire (for PRIME sites using SSCA). (PDF 396 kb)

## Abbreviations

CONSORT: Consolidated Standards of Reporting Trials
ICH: Intracerebral haemorrhage
MRC: Medical Research Council
PI: Principal investigator
PRIME: Promoting Recruitment using Information Management Efficiently
RCT: Randomised controlled trial
RESTART: REstart or STop Antithrombotics Randomised Trial
SPIRIT: Standard Protocol Items: Recommendations for Interventional Trials
SSCA: Scottish Stroke Care Audit
SSNAP: Sentinel Stoke National Audit Programme
SWAT: Study Within a Trial repository
