# Erratum to: Promoting Recruitment using Information Management Efficiently (PRIME): study protocol for a stepped-wedge cluster randomised controlled trial within the REstart or STop Antithrombotics Randomised Trial (RESTART)

**DOI:** 10.1186/s13063-017-1906-7

**Published:** 2017-03-28

**Authors:** Amy E. Maxwell, Martin Dennis, Anthony Rudd, Christopher J. Weir, Richard A. Parker, Rustam Al-Shahi Salman

**Affiliations:** 1 Centre for Clinical Brain Sciences, University of Edinburgh, Chancellor’s Building, 49 Little France Crescent, Edinburgh, EH16 4SB UK; 2 St Thomas’ Hospital, Westminster Bridge Road, London, UK; 3 Edinburgh Clinical Trials Unit and Centre for Population Health Sciences, Usher Institute of Population Health Sciences and Informatics, Medical School, University of Edinburgh, Teviot Place, Edinburgh, UK

## Erratum

In the original publication [1] figures S1 and S2 should have been part of the main text rather than additional files.

There were some minor typographical errors on pages 2 and 4 and in the backmatter.

Page 2 should have read: ‘There is a clear knowledge gap with regard to effective strategies aimed at recruiters’ ([2], pg 1) and page 4: ‘After the review, an email is sent to all the collaborators at the site, summarising what was discussed at the review, providing attendance certificates and giving instructions for running the relevant bespoke stroke audit data exports.’

The backmatter: Trial status: The first recruitment review took place in September 2015, and the last review in August 2016. The first 6-month follow-up took place in March 2016, with the last one in February 2017.’

The original article has been updated to rectify these errors.
