# Supplementary material for: Promoting Recruitment using Information Management Efficiently (PRIME): study protocol for a stepped-wedge cluster randomised controlled trial within the REstart or STop Antithrombotics Randomised Trial (RESTART)
Source: Trials. 2017 Mar 1;18:22. doi: 10.1186/s13063-016-1692-7 (PMC5331676; doi:10.1186/s13063-016-1692-7)
Supplement: Supplementary file 1 — Pre-recruitment review questionnaire. (PDF 409 kb) [file 13063_2016_1692_MOESM1_ESM.pdf]

THE UNIVERSITY of EDINBURGH

RESTART — REstart or STop Antithrombotics Randomised Trial

RESTART Pre-Recruitment Review Questionnaire

Thank you for agreeing to take part in a Recruitment Review. In preparation for it please take the time to complete and return this questionnaire to me.

Amy
Study Recruitment Co-Ordinator
Tel: 07490 691 430
Email: amy.maxwell@ed.ac.uk

Instructions for completion

- Please place a cross in the appropriate box e.g. Yes [X] No [ ]
- To start completing the form, please use your mouse to click in 'Centre Number' and use the tab key to move through the fields.
- If you do not have enough space for your answer please use the additional comments box at the end, clearly labelling it with the number of the question it relates to.

Centre Number

Centre Name

1. *(For English centres only)* **Has there been any impact on your site following the restructuring of the Stroke Research Network?** e.g. did you work solely in Stroke before the restructure, do you now work solely in Stroke following the restructure, if not what other specialities do you cover, has there been an impact on resources at your site etc.

2. (i) What proportion of In-Patients, who are suitable for follow-up, are seen in clinic after hospital discharge, (ii) who sees them and (iii) when?

RESTART Pre-Recruitment Review Questionnaire

3. Have you approached patients looked after by your stroke unit in the past to invite them back to clinic, with a view to recruit them to RESTART?

Yes No

Any comments;

4. Have you used the template invitation letter, disseminated with RESTART protocol v6, to invite potential RESTART patients to clinic?

Yes No

If Yes, how effective have they been?

5. Are your stroke audit data complete and accurate, to the best of your knowledge?

Yes No

Any comments;

6. (i) Who is the main contact for your hospital's stroke audit data, and (ii)what is their email address?

7. Do you routinely use the stroke audit data to identify potential patients for RESTART?

Yes **No**

Any comments;

8. What other sources of information do you have on patients looked after at your stroke unit in the past, which could be used to identify patients who might be eligible for RESTART? *(Please enter a 'X' in each relevant box)*

Screening logs **A database other than stroke audit**

Other – please specify;

9. Have you used any other methods to boost recruitment to RESTART? If yes, please give details below;

10. Have you found any barriers to finding suitable patients for recruitment to RESTART? If yes, please give details below;

Additional comments

Thank you *for taking the time to complete this questionnaire.*
