# Supplementary material for: Promoting Recruitment using Information Management Efficiently (PRIME): study protocol for a stepped-wedge cluster randomised controlled trial within the REstart or STop Antithrombotics Randomised Trial (RESTART)
Source: Trials. 2017 Mar 1;18:22. doi: 10.1186/s13063-016-1692-7 (PMC5331676; doi:10.1186/s13063-016-1692-7)

How to obtain customised RESTART reports from SSNAP (The Sentinel Stroke National Audit Programme)

REstart or STop Antithrombotics Randomised Trial

What you need to know:

These instructions explain how to use the SSNAP data submitted by your stroke service to download lists of your hospital's patients who may be suitable for the RESTART trial on the basis that:

a) They are aged ≥18 years now.
b) The latest data extract for SSNAP indicates that they are still alive.
c) They had intracerebral haemorrhage (ICH).
d) They are at least 24 hours after symptom onset.
e) They are registered with a General Practitioner (GP).
f) They were not reported to be taking an antiplatelet or anticoagulant drug after ICH.

There are two different reports called 'Restart With' and 'RESTART Without', both of which may contain suitable patients, but SSNAP data are unable to provide information about **ALL** of the RESTART eligibility criteria: the first contains patients who had comorbidities or a history of antithrombotic drug use before the ICH that suggest that they may have been on antithrombotic drugs at the time of ICH. These patients are **most likely** to be suitable for RESTART. The second contains patients who do not have a record of relevant comorbidities or antithrombotic drug use, but some of them may still be eligible for RESTART.

Please would you:

1) Involve a member of staff who is able to extract reports from the SSNAP database.
2) Adhere to your Trust's Information Governance procedures.
3) Follow the attached instructions to use the electronic plug-ins that we are providing to download the two exports of patients who are potentially eligible for RESTART.
4) Start with the first report, and then move on to the second.
5) Check that each patient is still alive and likely to be eligible using the patient's medical records, or contact with their general practitioner, before contacting them.
6) Contact patients who may be eligible using the template letters provided (that may be sent by the patient's hospital consultant or their GP), which have been approved by the research ethics committee.

What you need to do:

Attached with these guidelines are two electronic XML files (plug-ins) in a zipped folder. One called 'RESTART With' (this contains patients who had comorbidities or a history of antithrombotic drug use before the ICH) and one called 'RESTART Without' (this contains patients who do not have a record of relevant comorbidities or antithrombotic drug use). Please save both of these files in an accessible directory *(**NB do not open them** at this stage as they shall be opened in the SSNAP database)*, and follow the instructions below to produce the reports.

If you need help with any of these instructions please feel free to contact me.

Amy
Study Recruitment Co-Ordinator
Tel: 07490 691 430
Email: amy.maxwell@ed.ac.uk

How to obtain customised RESTART reports from SSNAP (The Sentinel Stroke National Audit Programme)

RESTART – REstart or STop Antithrombotics Randomised Trial

Step 1

➢ **'Sign in'** to SSNAP:

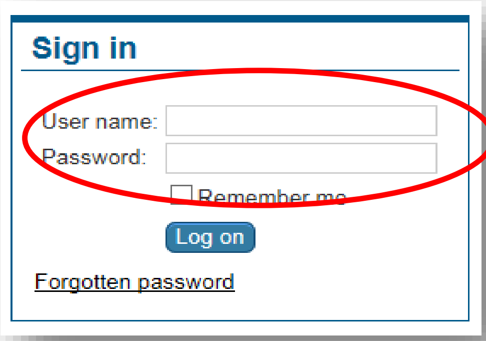

Step 2

➢ In the menu select **'Clinical'** then **'Custom export'**:

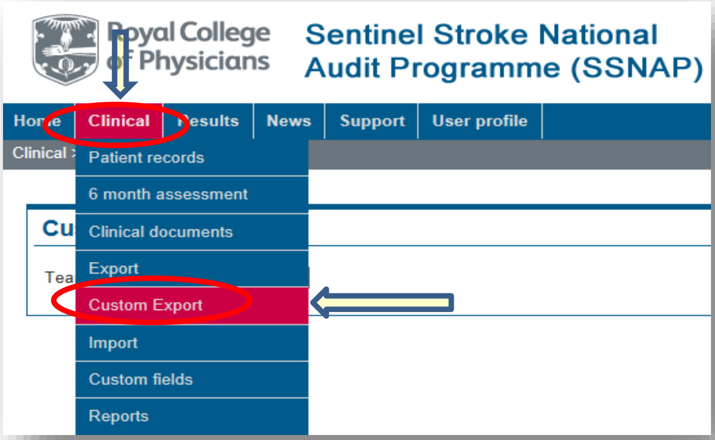

Step 3

➢ Select the team you wish to run the report for:

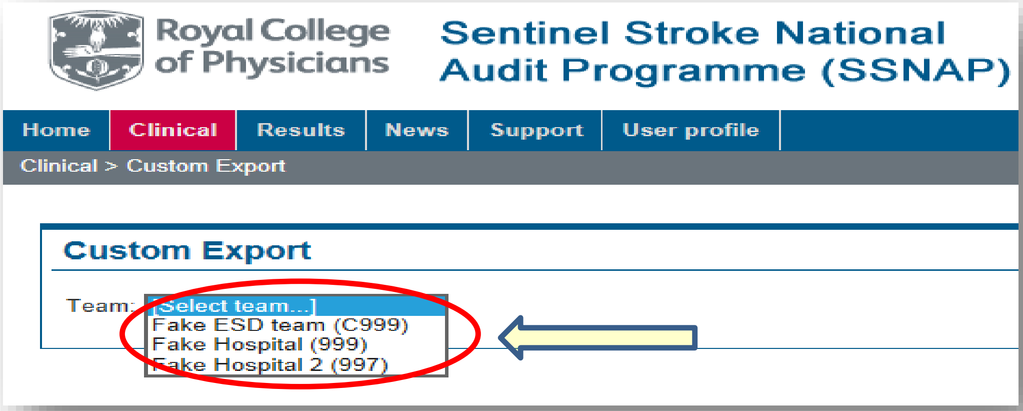

RE START? REstart or STop Antithrombotics Randomised Trial

Step 4

- Click the **'Browse'** button:

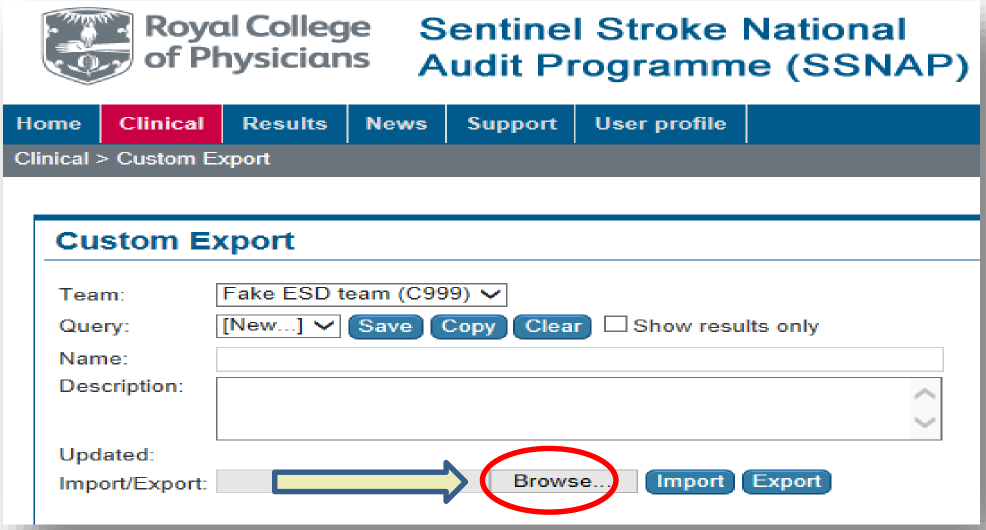

This will open up your file directory, find the SSNAP export XML files you saved and select one e.g. **'RESTART With'** and click on **'Open'**. You will now see the file name appear in the **'Browse'** box.

Step 5

- Now click on the **'Import'** button:

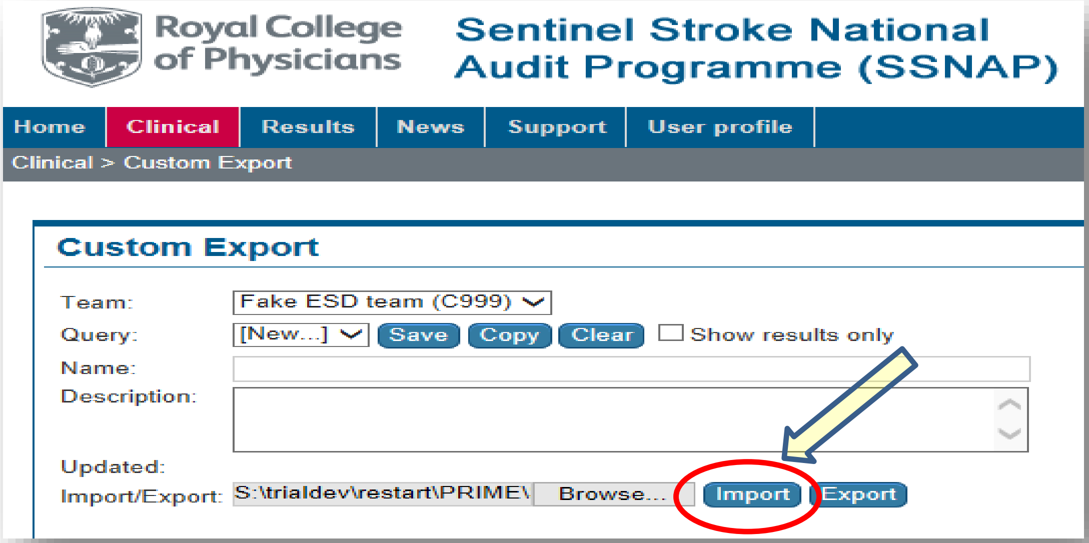

Step 6

- You will now see lists in the **'Results columns'** and **'Query conditions'**:

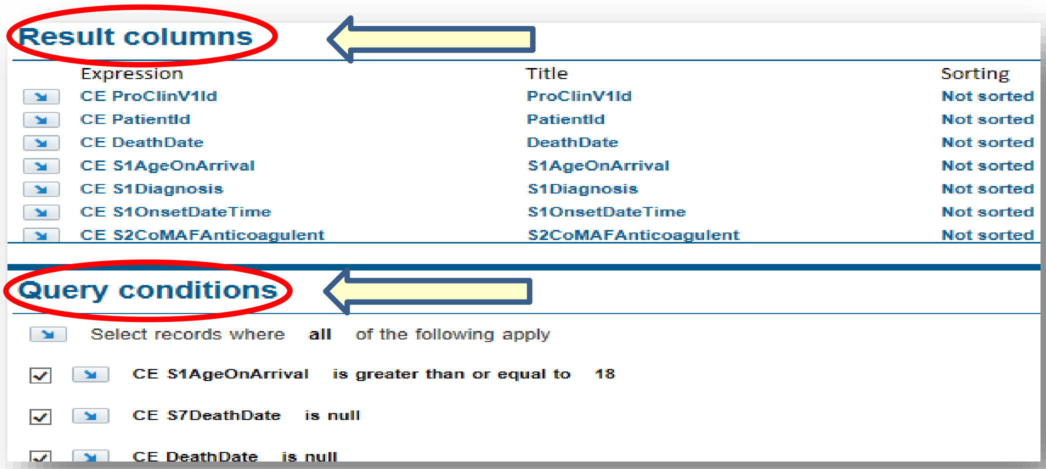

Step 7

- Ensure that the **'Onset date'** is more than 24hrs by checking and updating it (**N.B.** the calendar function may not work correctly depending on which version of Internet Explorer you are using, if this is the case manually enter it):

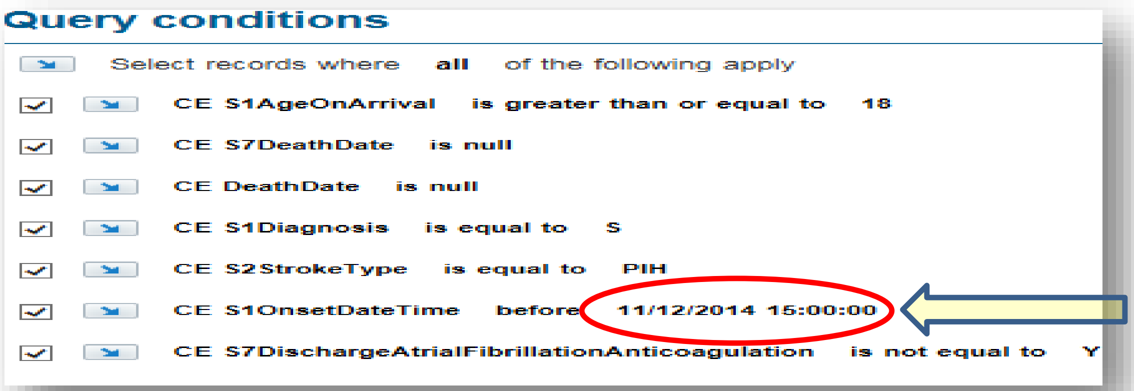

Step 8

- Click '**Update results**':

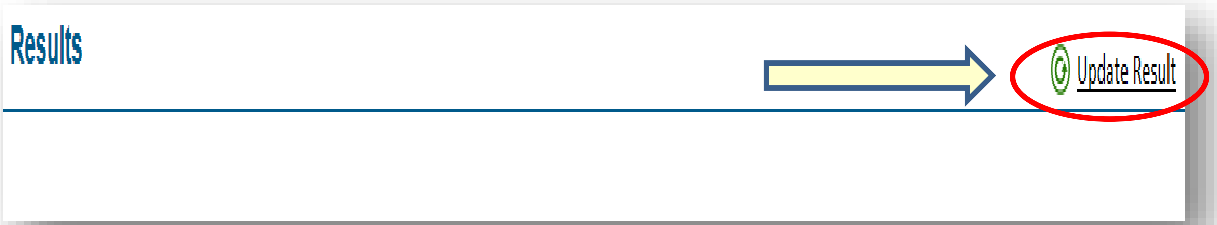

Step 9

- This will update the results box with the potential patients and allow you to export them to a CSV file by clicking on **'Export to CSV'**:

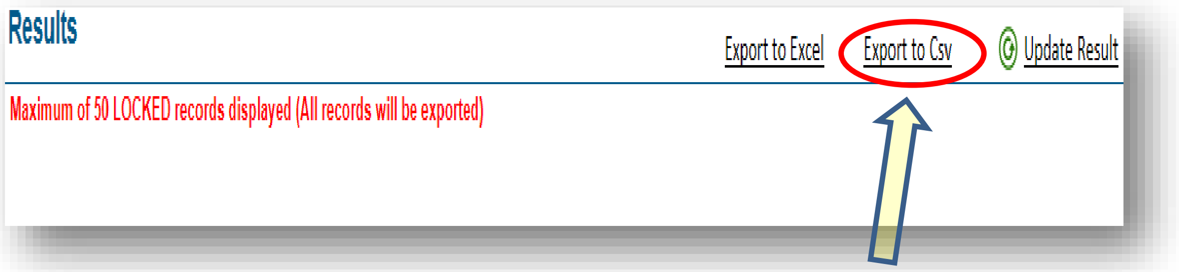

*You have now produced the report for patients **'RESTART With'** comorbidities so please save it and repeat the process for patients **'RESTART Without'** comorbidities. (We recommend that you save each report with the prefix of the date you downloaded it, to stop you accidently overwriting it next time you run the report.)*

To log out of the database

- Click on the **'Sign Out'** button in the top right hand side of the screen:

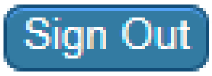

Supplement: Supplementary file 2 — SSNAP RESTART reporting instructions. (PDF 609 kb) [file 13063_2016_1692_MOESM2_ESM.pdf]
