# Supplementary material for: Promoting Recruitment using Information Management Efficiently (PRIME): study protocol for a stepped-wedge cluster randomised controlled trial within the REstart or STop Antithrombotics Randomised Trial (RESTART)
Source: Trials. 2017 Mar 1;18:22. doi: 10.1186/s13063-016-1692-7 (PMC5331676; doi:10.1186/s13063-016-1692-7)
Supplement: Supplementary file 3 — SSCA RESTART reporting instructions. (PDF 788 kb) [file 13063_2016_1692_MOESM3_ESM.pdf]

How to obtain customised RESTART reports from SSCA (The Scottish Stroke Care Audit)

What you need to know:

These instructions explain how to use the SSCA data submitted by your stroke service to download lists of your hospital's patients who may be suitable for the RESTART trial on the basis that:

a) They are aged ≥18 years now.
b) The latest data extract for SSCA indicates that they are still alive. **NB** *this is* ***retrospective data****, it is important to check* ***up to date patient records*** *to confirm patient status.*
c) They had intracerebral haemorrhage (ICH).
d) They are at least 24 hours after symptom onset.
e) They are registered with a General Practitioner (GP).
f) They were not reported to be taking an antiplatelet or anticoagulant drug after ICH.

There are two different reports called 'Inpatients Report 1' and 'Inpatients Report 2', both of which may contain suitable patients. The first contains patients who have suffered a haemorrhagic stroke and may have been on antithrombotic drugs at the time of ICH. These patients are **most likely** to be suitable for RESTART. The second contains patients who have suffered a haemorrhagic stroke and do not have a record of antithrombotic drug use at the time of ICH but still may be eligible for RESTART.

Please be aware: as SSCA does not collect **ALL** of the required data for RESTART eligibility criteria, you may find that there is a high proportion of patients included in the reports that have not been on antithrombotics prior to their stroke; you also may come across a small number of patients that have restarted antithrombotics already, and, as it is retrospective data there may be a large number of patients included that are now deceased, **therefore it is important that current patient records are checked before any contact is made.**

Please would you:

1) Involve a member of staff who is able to extract reports from the eSSCA database.
2) Adhere to your Health Board's Information Governance procedures.
3) Follow the attached instructions to produce the two reports (this will need to be completed by the eSSCA authorised user).
4) Start with the first report, and then move on to the second.
5) Check that each patient is still alive and likely to be eligible - using the patient's medical records, or contact with their general practitioner, before contacting them.
6) Contact patients who may be eligible using the template letters provided (that may be sent by the patient's hospital consultant or their GP), which have been approved by the research ethics committee.

How to produce the reports:

Follow the step by step instructions below to:

Check your **macro security settings** in excel.

Take an **extract of all the patients** from your Hospital and/or Health Board by using the SSCA database.

Copy all of the patients into the workbook I have sent you in order to create both reports.

Copy and Save the two reports into a separate workbook for your local RESTART team.

If you need help with any of the steps please feel free to contact me.

Amy
Study Recruitment Co-Ordinator
Tel: 07490 691 430
Email: amy.maxwell@ed.ac.uk

Macro settings

Before you produce the reports you need to ensure that your macro security setting in excel will allow you to run them. The way you do this will depend on the version of excel you are using; therefore please follow the appropriate step by step instructions below.

Excel 2003/2000

- Click on the **'Tools'** tab then **'Macro'**, **'Security'**, **'Medium'**, then **'OK'**.

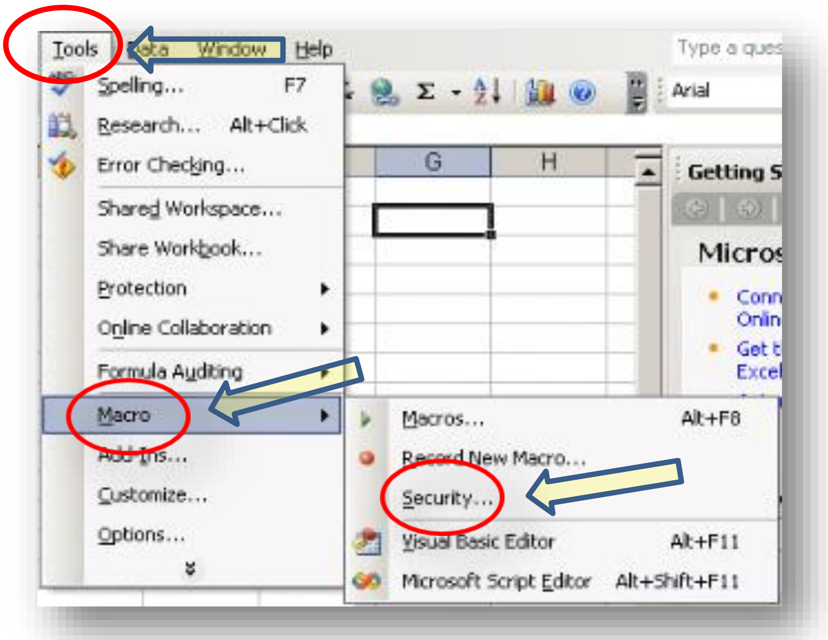

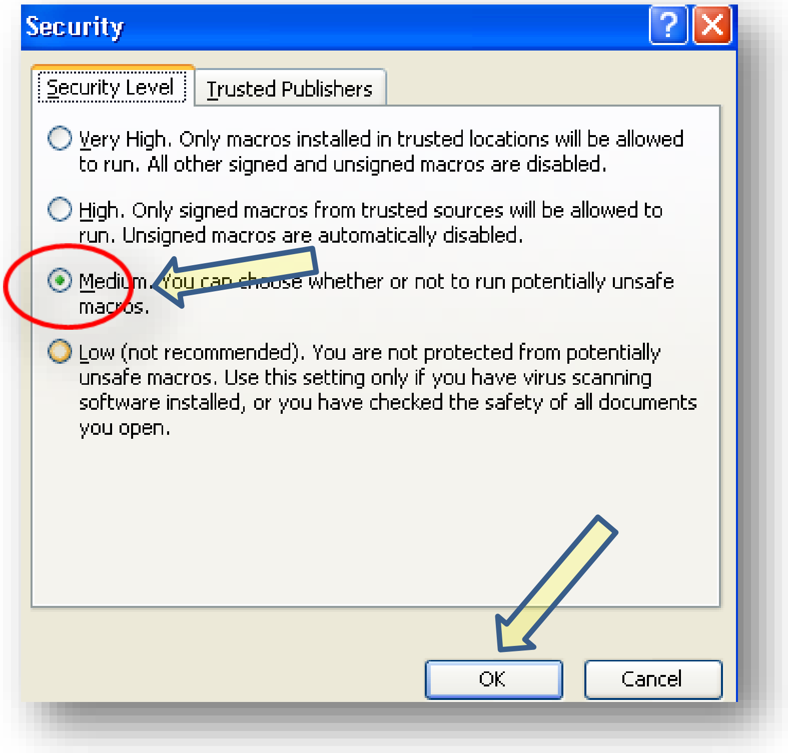

- Close the workbook for the changes to take affect. When you re-open the workbook you may get a security warning, click on **'Enable Macros'** so you will be able to run the reports.

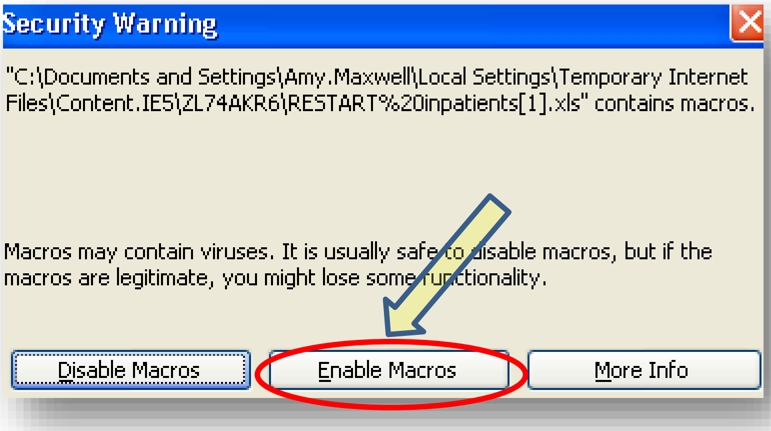

Excel 2007

- Click on the '**Home'** button (circular button top left of window) and then '**Excel Options'**, **'Trust Centre'**, **'Trust Centre settings'**, '**Macro Settings'** and then select **'Disable all macros with notification'** and **'OK'**.

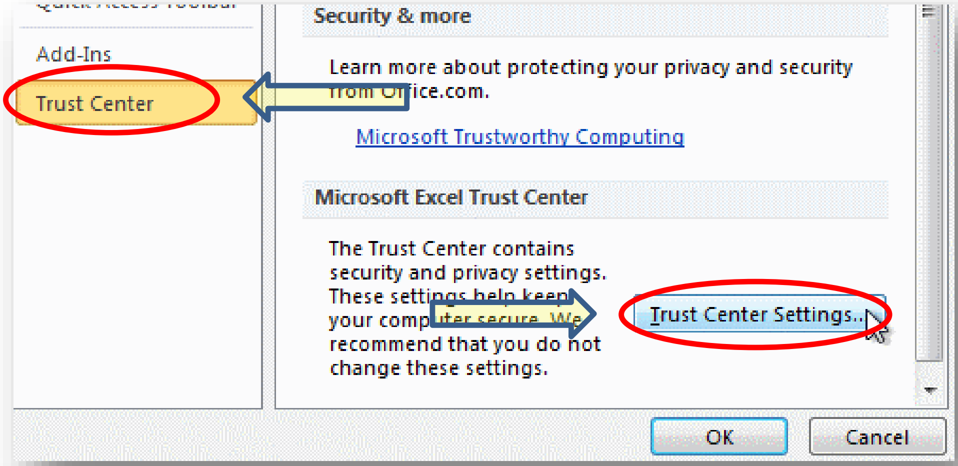

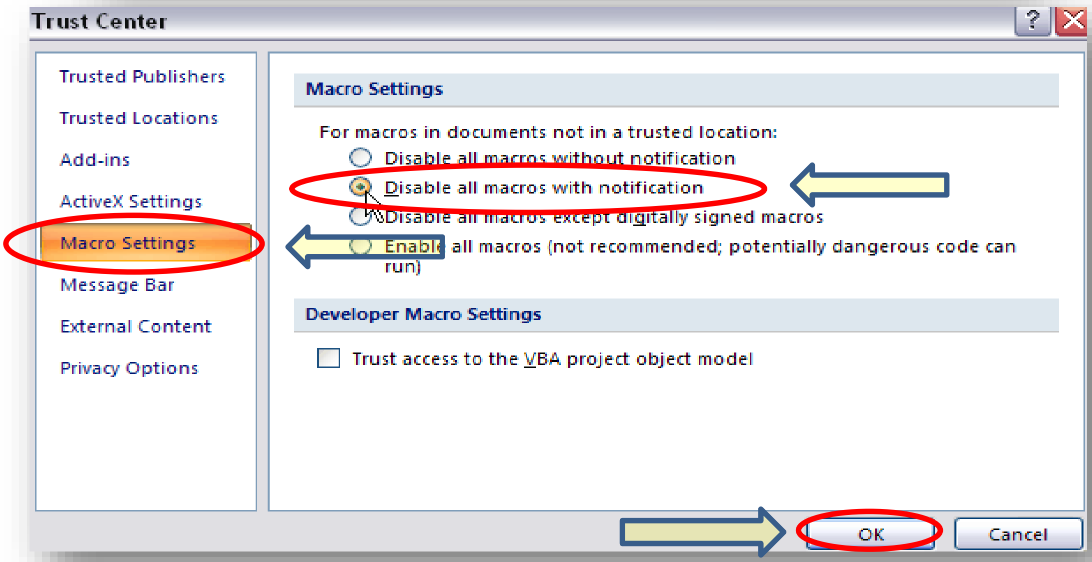

- Close the workbook for the changes to take affect. When you re-open the workbook you will see a security warning **'Macros have been disabled'**. Click **'Options'** then **'Enable this content'** in the security alert then **'OK'**.

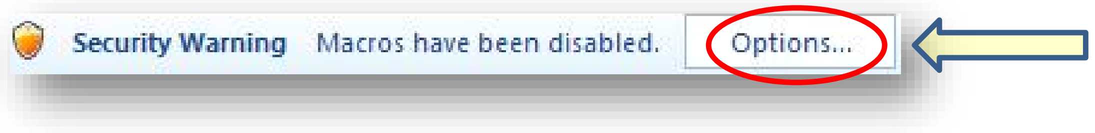

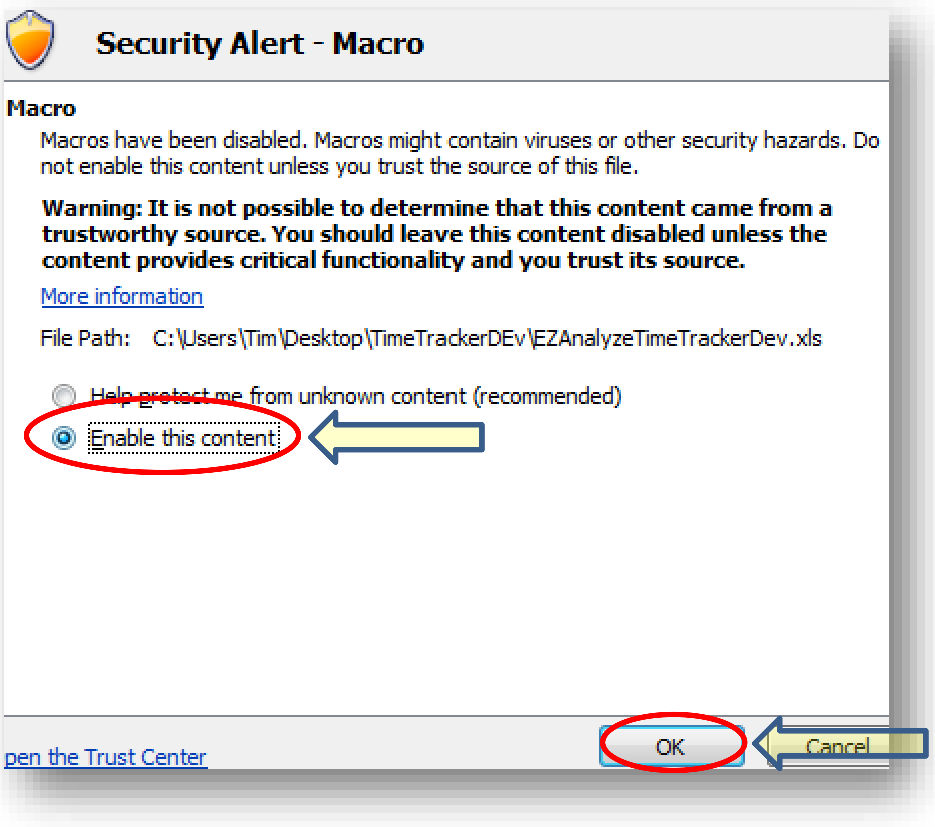

N.B. *Once you have completed the report you should revert your macro settings back to 'High' or 'Very High' regardless of which version of excel you are using.*

How to obtain customised RESTART reports from SSCA (The Scottish Stroke Care Audit)

Running the Reports

Step 1

Log into SSCA https://apex.nhsnss.scot.nhs.uk/apexp/f?p=saudit

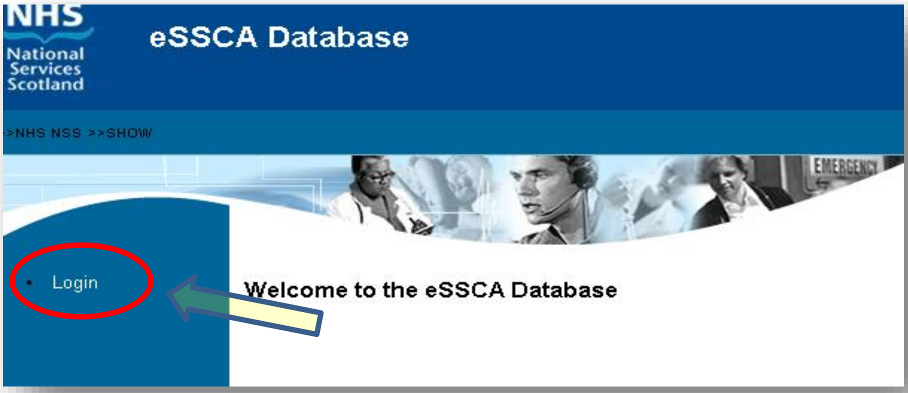

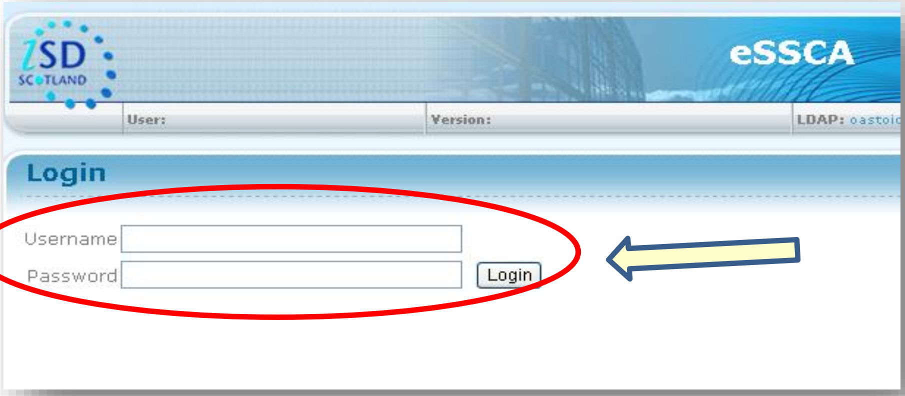

Step 2

➢ Select **'Reports'**:

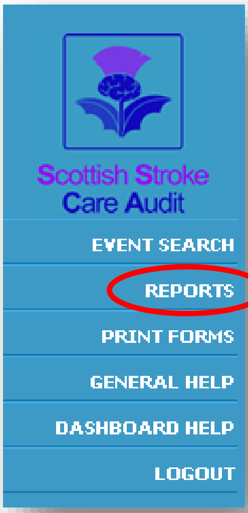

Step 3

- Click on **'Data Extract'**:

N.B. *this can take* ***some time*** *as eSSCA gathers all of the data for your health board.*

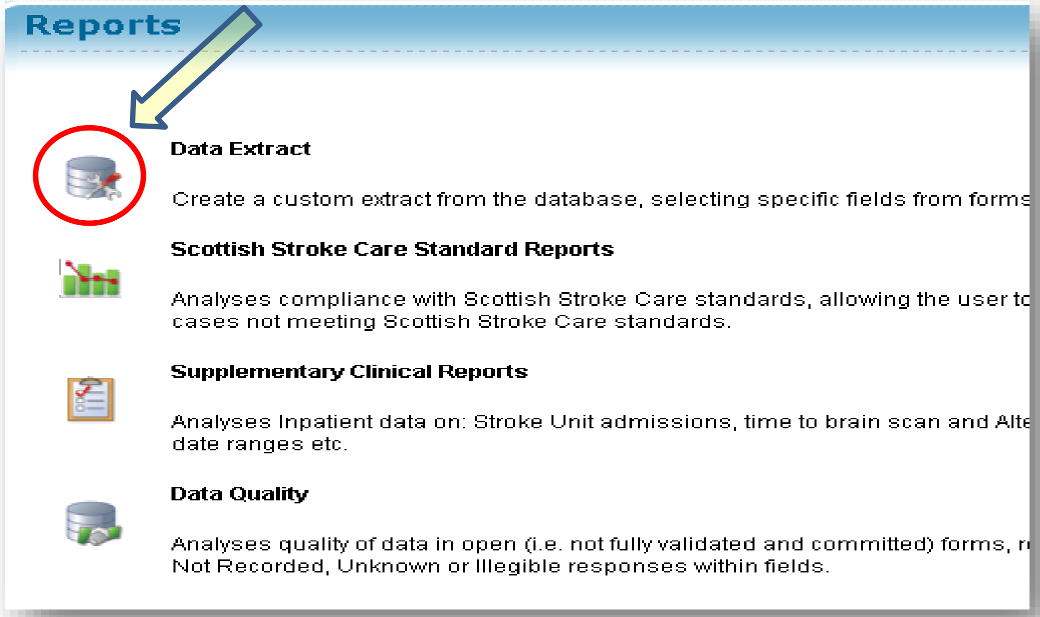

Step 4

- Click on **'Actions'** and then select **'Columns'**:

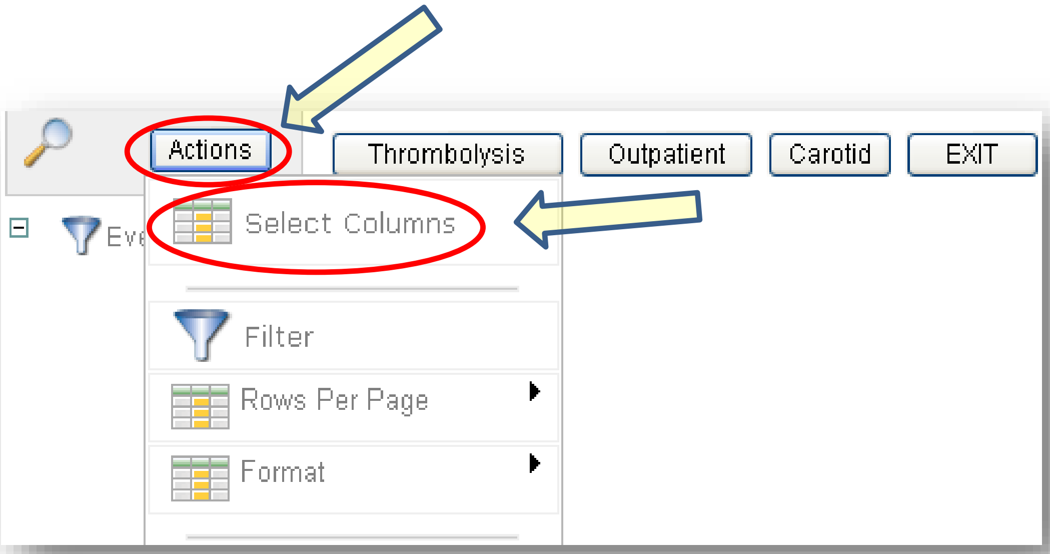

Step 5

- Click the **'double arrows'** pointing to the right to move all of the columns from the **'Do not Display'** box into the **'Display in Report'** box and then click **'Apply'**:

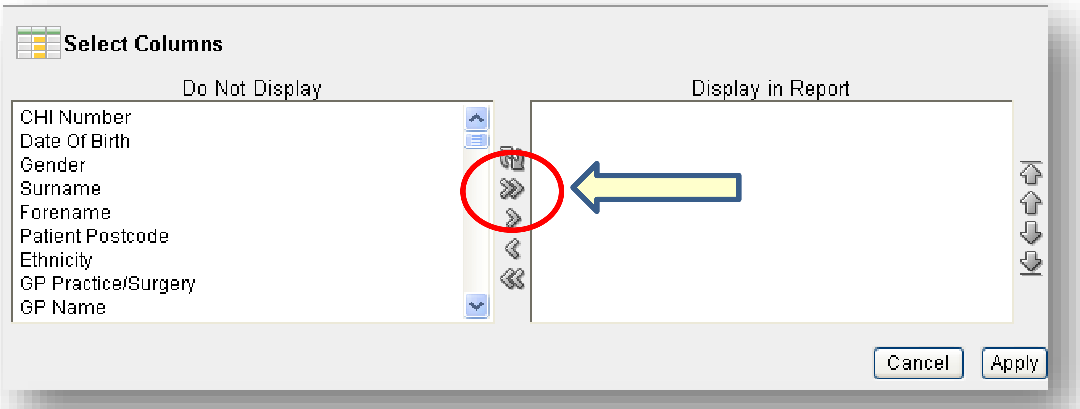

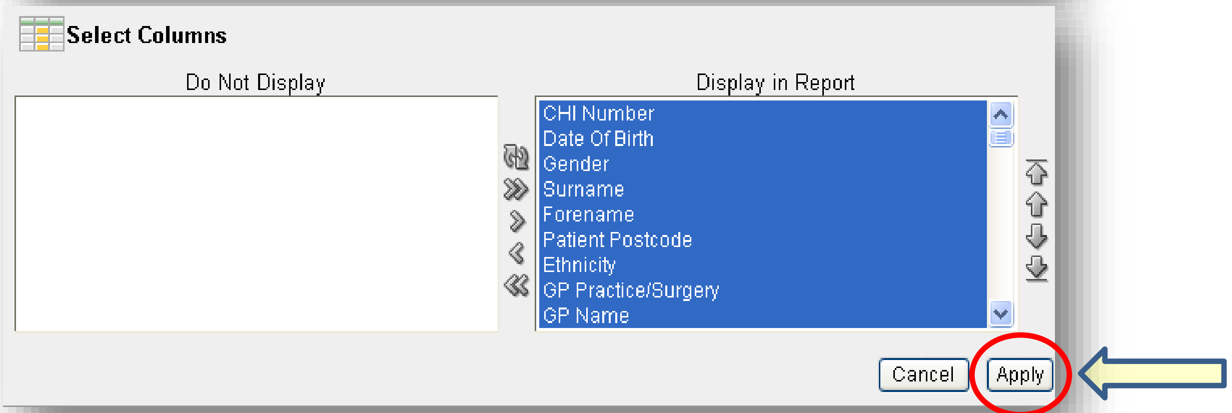

Step 6

- Click on the **'Actions'** again and select **'Download'**:

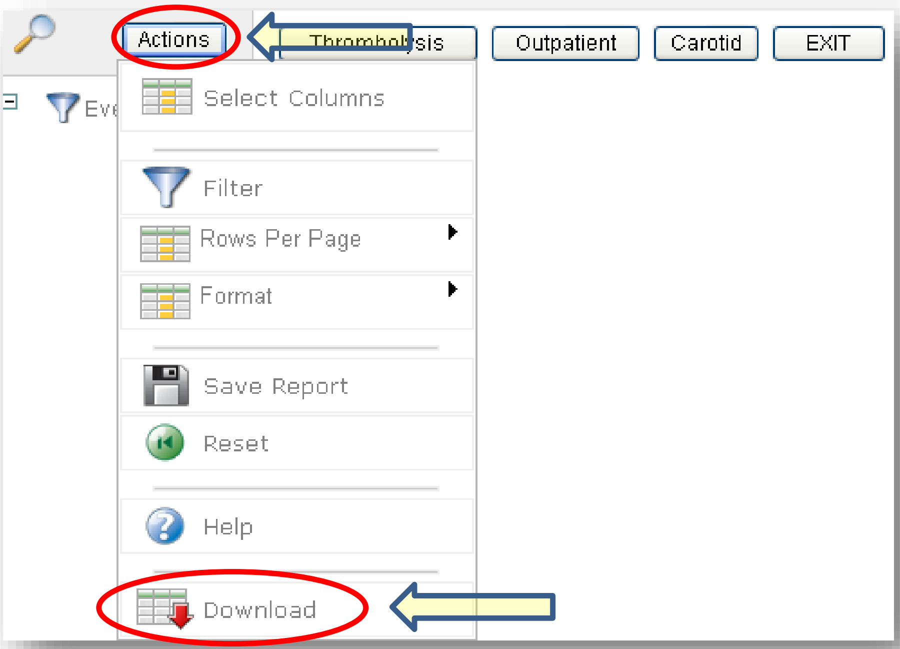

Step 7

➢ Click on the image above **'CSV'** to download the report:

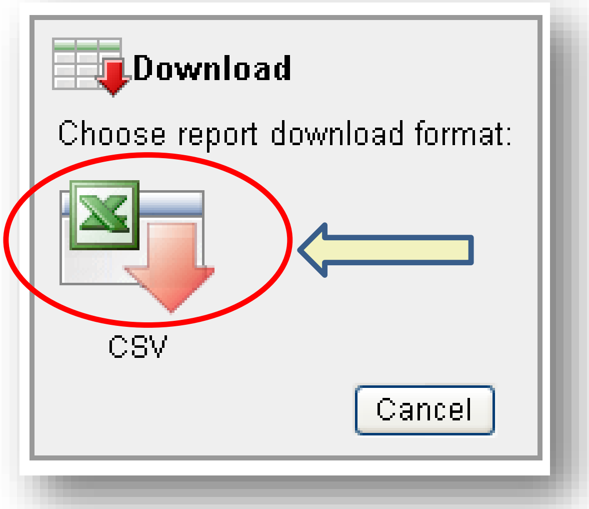

Step 8

➢ Once the **'File Download'** box appears click on **'Open'**:

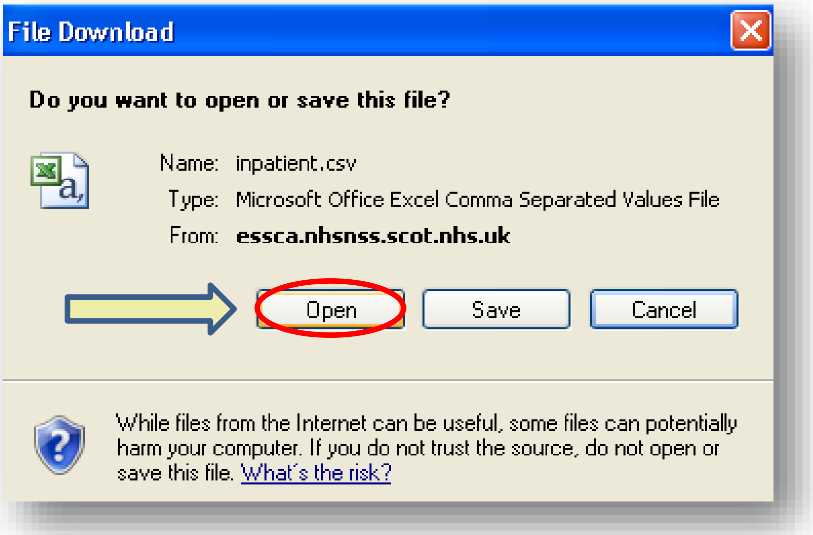

A CSV file will now open which will contain an extract of all the patients held in the database for your Hospital and/or Health Board. Log out of the SSCA database and then copy these patients into the workbook you have been sent (Step 9) to produce the two reports to be used by the RESTART team.

To log out of the SSCA database

- Click on the **'Exit'** button:

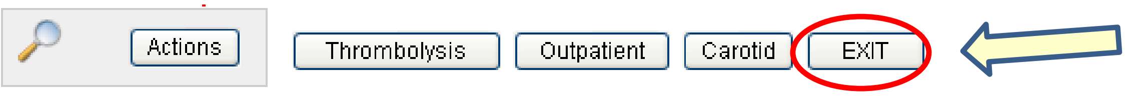

- Then click on **'Log Out'**:

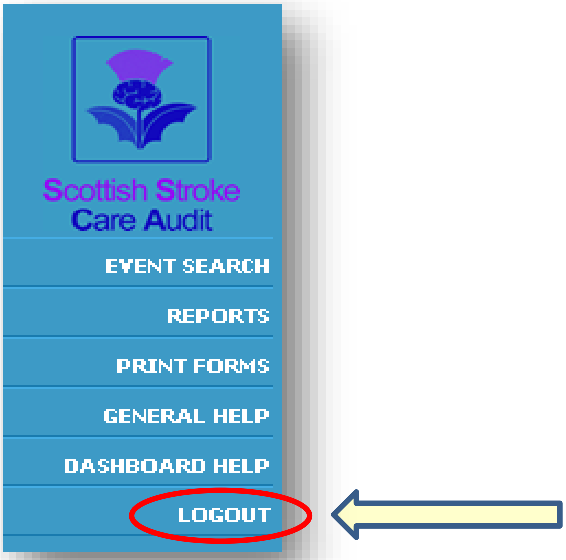

Step 9 – Running the reports

- Open the Excel workbook that was attached with these guidelines. Click on the **'Inpatient – Data'** tab and make sure it is blank:

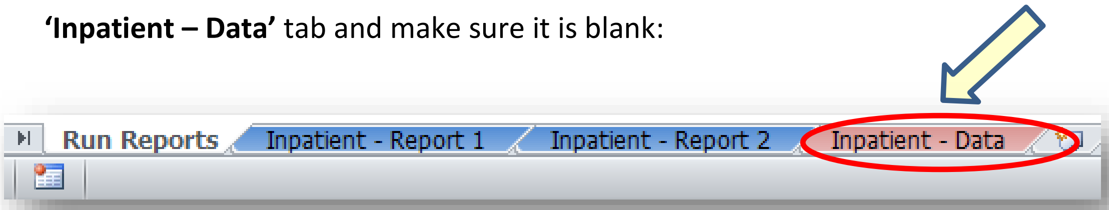

- Copy all of the data from the extract you produced (Step 7 & 8) by using **'Ctrl+A'** to select the data, then **'Ctrl+C'** to copy the data, and then **'Ctrl V'** to paste the data. Ensure that you paste it into **'cell A1'** of the **'Inpatient – Data'** tab. (If the data is not entered into cell A1 the report will not run properly).

- Before running the reports ensure that the tabs **'Inpatient - Report 1'** and **'Inpatient - Report 2'** are blank.

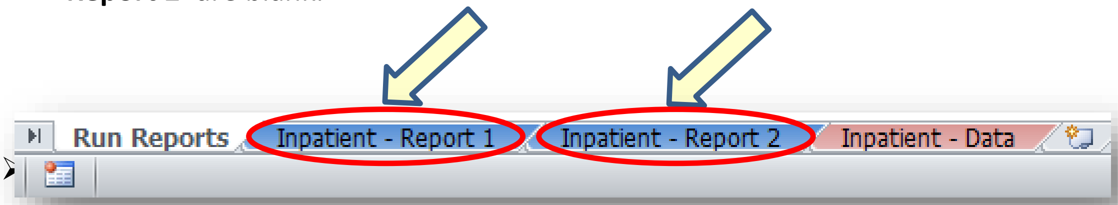

- Click on **'Run Reports'** tab and then on the **button 'Click to run Inpatient – Report 1'**. You will then be automatically directed to the **'Inpatient – Report 1'** tab and a box will pop up saying **'Inpatient Report 1 Complete!'**. Click on **'OK'**, and repeat the process for Report 2. You will now see the reports populated in their relevant tab. (If you want to re-run these reports at any time, please ensure you delete all the previous data first.)

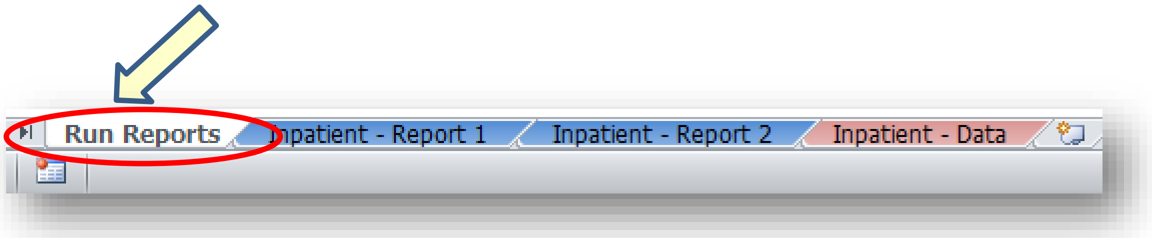

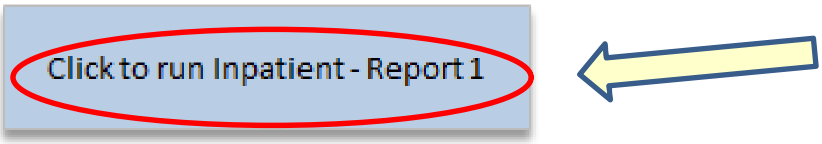

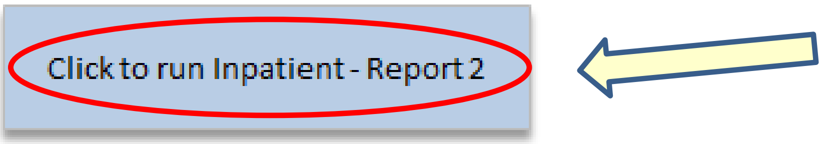

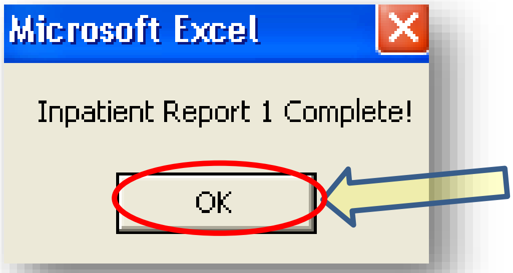

You have now produced the reports. We suggest that the two reports are copied into a separate workbook and sent to, or printed out for the RESTART co-ordinator (following local Information Governance Procedures), so they do not have a list of all the patients in the database.
