# Supplementary material for: Promoting Recruitment using Information Management Efficiently (PRIME): study protocol for a stepped-wedge cluster randomised controlled trial within the REstart or STop Antithrombotics Randomised Trial (RESTART)
Source: Trials. 2017 Mar 1;18:22. doi: 10.1186/s13063-016-1692-7 (PMC5331676; doi:10.1186/s13063-016-1692-7)
Supplement: Supplementary file 4 — Six-month follow-up review questionnaire (for PRIME sites using SSNAP). (PDF 396 kb) [file 13063_2016_1692_MOESM4_ESM.pdf]

THE UNIVERSITY of EDINBURGH

RESTART — REstart or STop Antithrombotics Randomised Trial

RESTART Recruitment Review 6 month Follow-up Questionnaire

Thank you for agreeing to have a 6 month follow up discussion. In preparation for it please take the time to complete and return this questionnaire to me.

Amy
Study Recruitment Co-Ordinator
Tel: 07490 691 430
Email: amy.maxwell@ed.ac.uk

Instructions for completion

- Please place a cross in the appropriate box e.g. Yes [X] No [ ]
- To start completing the form, please use your mouse to click in 'Centre Number' and use the tab key to move through the fields.

Centre Number ______

Centre Name ______

1. Since receiving your recruitment review, have you generated and used the audit data extract RESTART reports? *If 'Yes' go to question 2 If 'No' go to question 12*

Yes [ ] **No** [ ]

2. (i) How far back did you run the reports to, and

______

(ii) How many times have you run them?

______

	RESTART With	RESTART Without	Or Both
**3. How many patients did the reports identify?**			
**4. How many of these patients were eligible?**			
**5. How many of those eligible did you contact?**			
**6. Out of those contacted; how many responded?**			
**7. Out of those who responded; how many came back to clinic?**			
**8. Out of those who responded; how many declined?**			
**9. How many that returned were randomised?**			

Any questions, contact Amy Maxwell: Amy.Maxwell@ed.ac.uk 07490 691 430

**10. Do you think that the reports were useful in identifying potentially eligible patients?**	Strongly agree	Agree	Neutral	Disagree	Strongly disagree
	☐	☐	☐	☐	☐

11. Have you had any (i) problems running or using the reports e.g. received any complaints about them, had lack of time/resource to use them, or (ii) think there are any disadvantages with using the reports?

Yes ☐ *If 'Yes' please provide details below* **No** ☐

12. If you didn't use the bespoke reports (Q 1) What were the barriers that prevented you from using them?

13. Are there any improvements you would like to suggest for the reports?

Thank you *for taking the time to complete this questionnaire.*
